# HLAEquity: Examining biases in pan-allele peptide-HLA binding predictors

**DOI:** 10.1016/j.isci.2023.108613

**Published:** 2023-12-02

**Authors:** Anja Conev, Romanos Fasoulis, Sarah Hall-Swan, Rodrigo Ferreira, Lydia E. Kavraki

**Affiliations:** 1 Department of Computer Science, Rice University, Houston, TX, USA

**Keywords:** Immune system, Computational bioinformatics, Machine learning, Human Geography

## Abstract

Peptide-HLA (pHLA) binding prediction is essential in screening peptide candidates for personalized peptide vaccines. Machine learning (ML) pHLA binding prediction tools are trained on vast amounts of data and are effective in screening peptide candidates. Most ML models report the ability to generalize to HLA alleles unseen during training ("pan-allele" models). However, the use of datasets with imbalanced allele content raises concerns about biased model performance. First, we examine the data bias of two ML-based pan-allele pHLA binding predictors. We find that the pHLA datasets overrepresent alleles from geographic populations of high-income countries. Second, we show that the identified data bias is perpetuated within ML models, leading to algorithmic bias and subpar performance for alleles expressed in low-income geographic populations. We draw attention to the potential therapeutic consequences of this bias, and we challenge the use of the term “pan-allele” to describe models trained with currently available public datasets.

## Highlights

• PHLA binding data have HLAs more common in high-income populations than low-income ones
• HLA bias in training data affects the pan-allele models’ performance
• Pan-allele predictors have lower accuracy for HLAs not found in training datasets
• Pan-allele predictors have lower accuracy for HLAs expressed in lower income populations

## Graphical abstract

**Figure:**
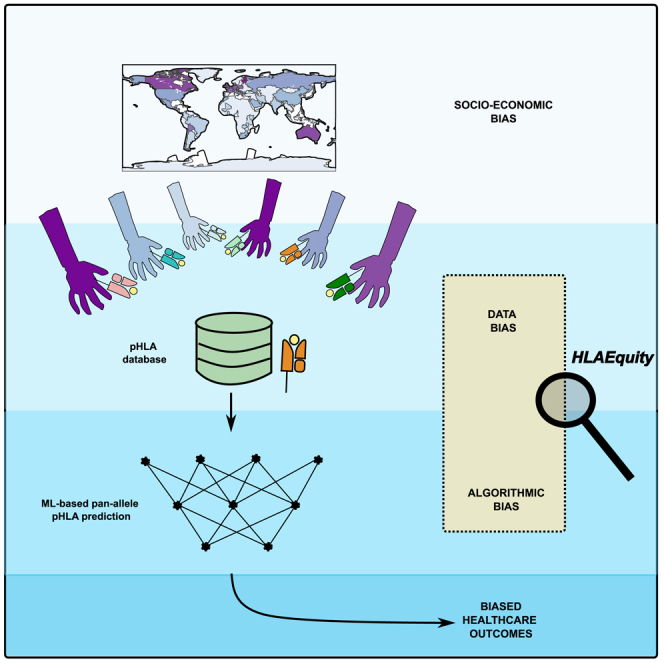

## Introduction

The adaptive cellular immune response is a vital aspect of the human immune system, seeking to destroy infected or cancerous cells. A major component of the adaptive immune response in humans is the peptide-human leukocyte antigen (pHLA) complex, which consists of a class I HLA receptor and a bound peptide derived from the proteasomal cleavage of intracellular proteins. Circulating T cells recognize and respond to HLAs presenting a foreign peptide stemming from a viral or a cancer protein. Peptides that bind to HLAs are targets for therapeutics ranging from cancer immunotherapy to viral vaccines. Predicting binding affinity between target peptides and HLAs is a crucial step in developing effective therapeutics.^1^

The task of predicting pHLA binding affinity is challenging. Genes encoding HLA receptors are among the most variable genes in the human genome, with over 25,000 identified alleles across the global population. Additionally, the number of potential peptide targets is large and difficult to experimentally screen. However, high-throughput mass spectrometry brought about increasing amounts of pHLA binding data. These data opened the door for *in silico* pHLA binding affinity prediction and the development of machine-learning (ML)-based tools, with the latest approaches adopting neural network architectures.^2^^,^^3^ Several ML models in the current literature provide pHLA binding affinity predictions for any HLA allele, even when the allele is absent during the training process. The authors of these models refer to them as “pan-allele” models.^2^^,^^3^ The promise of pan-allele predictions has great therapeutic significance, as it enables prediction for any HLA expressed in a patient. The early models were proclaimed as a technology that will enable individualized immunotherapy.^4^ Today, pan-allele prediction models are a significant component in immunotherapy pipelines.^5^^,^^6^^,^^7^ A recent survey identified 27 different methods for pHLA binding affinity prediction^8^; 20 out of 27 methods claim to be pan-allele while 17 out of 20 pan-allele methods utilize ML and neural network approaches (see also Table 1). The field strongly leans toward the ML-based pan-allele prediction paradigm.

**Table 1:** List of pan-allele pHLA binding affinity predictors compiled by a recent comprehensive review by *Wang* et al with the addition of the number of citations (queried from the PubMed library July 2023)

Predictor name^8^	Algorithm	Software available	Citations	Year	Reference
**NetMHCpan 4.1**	**FFNN**	**Y**	**753**	**2020**	Reynisson et al.^9^
**MHCflurry 2.0**	**FFNN**	**Y**	**491**	**2018, 2020**	O’Donnell et al.,^10^ O’Donnell et al.^2^
NetMHCcons	Consensus	N	340	2012	Karosiene et al.^11^
MixMHCpred	Scoring function	Y	314	2017, 2018	Bassani-Sternberg et al.,^12^Gfeller et al.^13^
PickPocket	Scoring function	Y	198	2009	Zhang et al.^14^
NetMHCstabpan	FFNN	Y	154	2016	Rasmussen et al.^15^
ConvMHC	CNN	Y	94	2017	Han et al.^16^
DeepHLApan	GRU+Attention	Y	72	2019	Wu et al.^17^
PSSMHCpan	Scoring function	Y	64	2017	Liu et al.^18^
MHCSeqNet	GRU	Y	59	2019	Phloyphisut et al.^19^
ACME	CNN	Y	55	2019	Hu et al.^20^
DeepSeqPan	CNN	Y	53	2019	Liu et al.^21^
TransPHLA	Multi-head self-attention	Y	38	2022	Chu et al.^22^
Anthem	AODE	Y	30	2021	Mei et al.^23^
MHCAttnNet	LSTM+Attention	Y	26	2020	Venkatesh et al.^24^
DeepAttentionPan	CNN+Attention	Y	12	2021	Jin et al.^25^
MATHLA	LSTM+Attention	Y	10	2021	Ye et al.^26^
HLAB	XGBoost, KNN, SVM, NB, LR, DTree, Bagging	Y	9	2022	Zhang et al.^27^
DeepNetBim	CNN+Attention	Y	8	2021	Yang et al.^28^
Seq2Neo	CNN	Y	5	2022	Diao et al.^29^

In the field of ML, it is widely recognized that models can demonstrate various forms of bias. As the models are deployed in real-world applications, this phenomenon can lead to disparate impacts.^30^ Biased facial recognition software showed discrimination based on race and gender.^31^ Decision-making algorithms deployed in crime prediction, credit lending, and hiring can perpetuate racial bias and injustice.^32^^,^^33^ The same issues arise with ML applications in healthcare. A risk assessment algorithm was found to misassign sick Black patients with the same low level of risk as less sick White patients.^34^ There are also issues related to ML bias in genomic-driven cancer treatments, as most of the sequenced patients in The Cancer Genome Atlas project are of European ancestry, while people with other ancestries are underrepresented.^35^

Focusing on ML models in healthcare, Norori et al. categorized different perspectives of bias as human, data, and algorithmic bias.^36^ Human bias refers to the individual biases, societal prejudices, and power imbalances that affect every human. Because humans create the data and the algorithms, our biases have a direct effect on what we create regardless of intent. Data bias refers to imbalanced data that may not be representative of the relevant portion of the human population. Lastly, algorithmic bias refers to how algorithms enforce the biases in the data. In healthcare, this translates to ML models that could give misguided predictions on specific geographic populations, affecting the efficacy of treatments that they might receive. Algorithmic bias includes the training criterion chosen for an ML model, as well as the way existing imbalances in the data (e.g., class imbalance) are handled during training.^36^

In this work, we investigate data and algorithmic bias in current pan-allele pHLA binding affinity prediction models. First, we find bias within publicly available pHLA datasets. Using the population coverage metric, we clearly see that the available peptide-HLA datasets do not equally represent different geographic populations. Moreover, by using the four different income classification levels defined by the World Bank, we associate the inequalities found in the calculated allele population coverage with income inequalities between nations. Next, we look at the algorithmic bias in two popular pan-allele pHLA binding predictors. We discover that the algorithms perpetuate the data bias, leading to differences in model performance across alleles. Due to this algorithmic bias, populations in lower income countries could benefit less from the ML predictions of the pan-allele models than populations in higher income countries, in regards to therapeutic efficacy. Ultimately, we question the use of the term “pan-allele” to describe a pHLA binding predictor. Our aim is to raise consciousness about the possible impact that bias can have in pHLA binding predictors, and, ultimately, in immunoinformatics and immunotherapy research.

## Results

### Skewed allele representation in pHLA training datasets reveals data bias disadvantaging low-income populations

**Figure 1:**
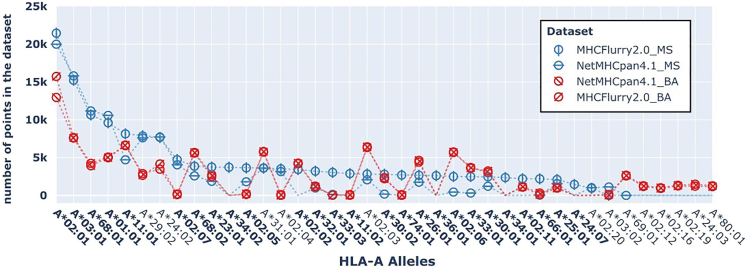
HLA-A allele frequencies in each of the training datasets (i.e., MHCFlurry2.0_BA, NetMHCpan4.1_BA, MHCFlurry2.0_MS, NetMHCpan4.1_MS) Allele codes are indicated on the x axis while the number of points in the dataset for each allele is indicated on the y axis. Allele codes are bolded if the respective number of data points in MHCFlurry2.0_MS is higher than the number of data points in NetMHCpan4.1_MS.

First, we investigate the distribution of alleles in the training datasets of the NetMHCpan4.1^3^ and MHCFlurry2.0^5^ models (Figures 1, S1, and S2). Note that mass spectrometry (MS) datasets (blue) have more data than binding affinity (BA) datasets (red). This is expected as the MS experiments have higher throughput. Overall, each dataset contains data for a limited number of alleles as compared to over 25,000 present in the human population and the allele distributions have a “long tail.” In particular, there are less than 25 alleles that are represented with more than 5,000 data points in the datasets. We notice an overrepresentation of the A∗02:01 and A∗03:01 alleles. Previous literature^37^ shows that alleles A∗02:01 and A∗03:01 are most prevalent in Caucasian populations while A∗11:01 and A33:03 are prevalent in Asian populations and A∗23:01 and A∗30:02 are most common in African populations. As expected, since both NetMHCpan4.1 and MHCFlurry2.0 collect their data from the Immune Epitope Database (IEDB),^38^ BA datasets have similar allele content (i.e., red markers align). However, MHCFlurry2.0_MS has more data as compared to NetMHCpan4.1_MS for a few alleles (for example, A∗11:01, A∗34:02, B∗40:02, C∗12:02 among others). These alleles are outlined in bold in Figures 1, S1, and S2 and for them, the blue lines in the plot diverge. In particular, MHCFlurry2.0_MS includes recently collected by Sarkizova et al.^37^ targeting most of the human population and specifically targeting some of the previously underrepresented alleles.

**Figure 2:**
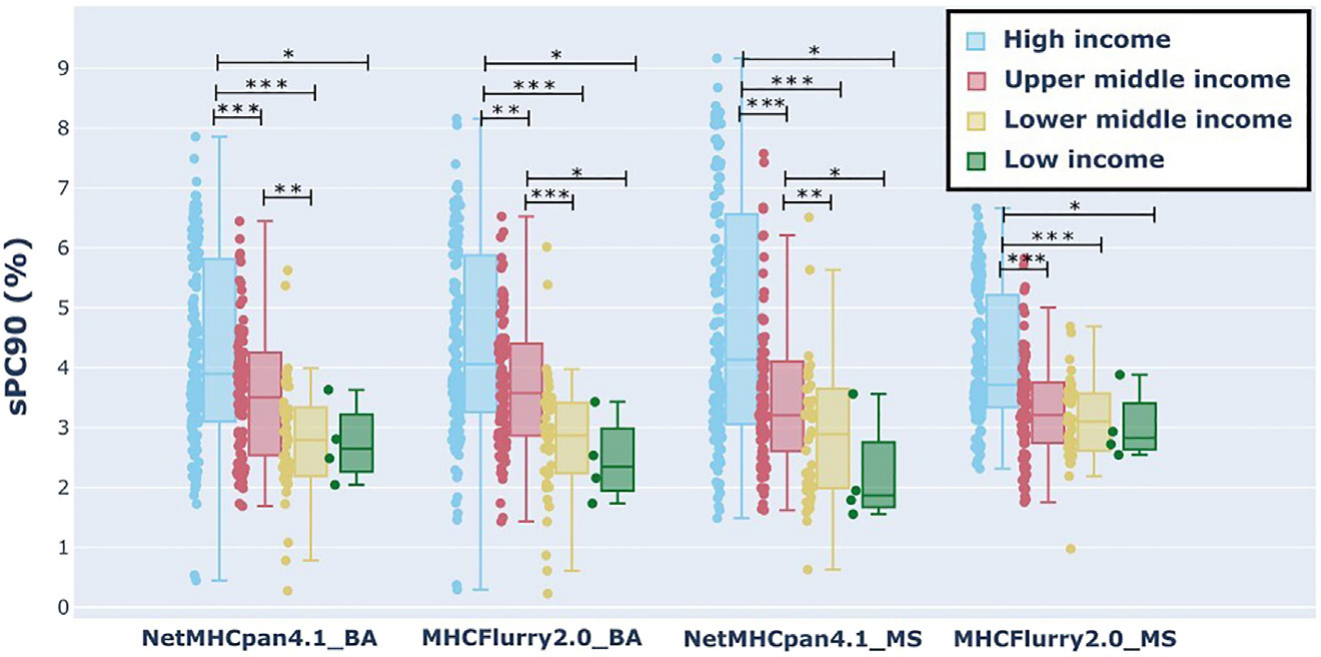
Scaled population coverage (sPC90) indicated on the y axis of training datasets indicated on the x axis Each point corresponds to a particular geographic population and points are grouped into boxplots based on the income level of the population’s country. The difference between the sPC90 distributions is evaluated with one-way ANOVA and the one-sided KS test. ∗p < 0.05; ∗∗p < 0.01; ∗∗∗p < 0.001.

Second, we quantify how the allele content of each dataset relates to the allele contents of specific geographic populations (Figure 2). We calculate the scaled population coverage (sPC90)^39^ for each dataset across all geographic populations contained in the Allele Frequency Net Database (AFND).^40^ The higher values of sPC90 indicate a better representation of the population within the dataset. We group the results based on the income level of the country of origin (Figure 2). We see a clear imbalance in terms of population coverage across different income levels. We highlight the statistical significance in the distributions of sPC90 across populations of different income levels. All datasets are biased toward the countries with higher income levels and on average they have higher sPC90 coverage for those populations. Note that the difference in sPC90 across the income categories is smallest for MHCFlurry2.0_MS (the boxes are closer together and the green low-income box is higher than for other datasets). The data recently sampled by Sarkizova et al.^37^ for underrepresented alleles and included in the MHCFlurry2.0_MS could be narrowing this difference down. Note that the high and the higher middle-income populations have a high deviation of the sPC90 scores. This is especially evident in countries with a high diversity of the ancestries of the populations within the country. For example, when we divide the US populations by their ancestry (Figure S3), we see that different ancestries are unequally represented.

### Pan-allele algorithms produce less accurate predictions for alleles from low-income populations

We assess whether the notion of algorithmic bias, as defined by Norori et al.,^36^ exists in popular pan-allele pHLA binding prediction tools. Algorithmic bias could be caused by training bias or the imbalance that occurs by having an uneven number of data points corresponding to each allele. In the pHLA binding prediction task, the algorithmic bias would translate to having vastly unequal prediction performance for alleles that are expressed in different geographic populations. We tested both NetMHCpan4.1 and MHCFlurry2.0, two widely used pan-allele neural network-based pMHC binding predictors, on the dataset from Pyke et al.^41^ (see STAR methods). Ideally, we would like to see NetMHCpan4.1 and MHCFlurry2.0 performing equally well on all HLA alleles (with both positive predicted value [PPV] and fraction of observed peptides [FOOP] being high). This would ensure that predictions of these models are accurate and can be used in downstream applications and therapeutics, independently of a patient’s geographic origin or allele expression.

**Figure 3:**
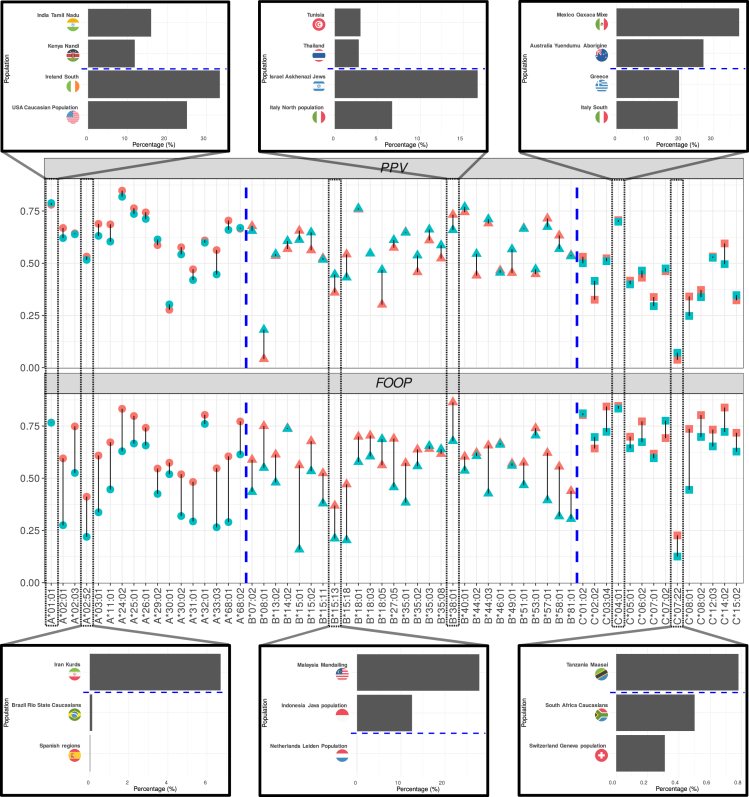
PPV (first row) and FOOP (second row) results for MHCFlurry2.0 (points in red) and NetMHCpan4.1 (points in blue) The x axis corresponds to different alleles in the dataset. The y axis corresponds to either the computed PPV value or the computed FOOP value. Different point shapes correspond to different HLA loci (A, B, C), separated by blue dashed lines. The top and bottom barplots correspond to alleles that have very good or very bad PPV and FOOP scores on average, respectively. For each allele, we plot low-income populations (above the blue dashed line) and higher income populations (below the blue dashed line) that express this allele the most.

Performance for MHCFlurry2.0 and NetMHCPan4.1 can be seen in Figure 3. Both MHCFlurry2.0 and NetMHCPan4.1 perform differently across different alleles. Moreover, we see that the fluctuations in performance mostly follow the same pattern for both MHCFlurry2.0 and NetMHCpan4.1, indicating that the two tools mostly succeed on the same alleles and fail on the same alleles too. Nevertheless, both methods fail to perform equally well on all alleles, given the big fluctuations in per-allele performance.

Furthermore, we identify alleles that both MHCFlurry2.0 and NetMHCpan4.1 succeed or underperform in terms of PPV and FOOP. The allele HLA-A∗02:52, for which the models are underperforming, especially in terms of FOOP, was previously identified to be prominent in Iranian Kurdish populations,^41^ and at the same time, it is not prominent in higher income countries or populations. It is also an allele that did not exist in the datasets that were used by NetMHCpan4.1 and MHCFlurry2.0 for training, indicating that pan-allele ML models may well underperform for alleles not previously seen. On the contrary, the allele HLA-A∗01:01, previously found to be expressed in high percentage in European and North American populations,^37^ performs very well, both in terms of PPV and FOOP. Similarly, the allele HLA-B∗15:13 is a low-performing allele for both pHLA binding prediction tools and is mostly expressed in upper/lower middle-income countries like Malaysia or Indonesia, but it is non-existent in higher income countries and populations. On the contrary, allele HLA-B∗38:01 is much more prominent in high-income countries (examples here are Israel and Italy) than in countries of low/middle income (examples here are Tunisia and Thailand). Similar patterns arise when examining other high/low-performing allele pairs, with very few notable exceptions, such as the HLA-B∗08:01, an allele expressed mostly in higher income populations, but with remarkably low PPV.

## Discussion

In this study, we inspect data and algorithmic bias in the pHLA binding prediction pipeline. We examine the content of different training datasets and identify the lack of alleles corresponding to populations in lower income countries. For example, there are many data points associated with the alleles prevalent in European populations (i.e., A∗02:01), while there are fewer points for the alleles prevalent in the African (i.e., A∗23:01) or Asian (i.e., A∗11:01) populations (Figure 1). This finding is quantified with the population coverage metric (sPC90) in Figure 2 and it is clear that the populations in higher income countries are better represented by the datasets. We showcase how the pan-allele algorithms accumulate and perpetuate identified data biases. We specifically show that state-of-the-art pHLA binding predictors underperform on alleles expressed in populations in lower income countries (i.e., Iranian Kurds, Malaysia Mandailing, Tanzania Masai populations). Ultimately, because these algorithms do not perform well on all alleles, they should not be described as pan-allele, as the term falsely implies that they will provide good predictions for all alleles. Our findings are significant for the future development of medical treatments on at least two levels.

At one level, we can take these results to highlight potential disparate impacts when it comes to the usage of these datasets and models for developing medical treatments for different geographic populations. Through our analysis, we find that MHCFlurry2.0 and NetMHCpan4.1 perform poorly on some alleles while performing well on others. More importantly, the models have superior performance for populations from high-income countries for which alleles are highly represented in the datasets, as compared to populations from low-income countries that are not well represented by the alleles in the datasets. When a tool does not perform well on certain alleles, the therapeutics that are developed using that tool may not perform well on individuals who have those alleles. Therefore, there is a danger of developing sub-par peptide vaccines or T cell-based immunotherapy protocols for certain populations from lower income countries. This distinction would only help exacerbate the long history of inequity that has existed when it comes to medical treatment for groups in higher income countries than for groups in lower income countries.

**Figure 4:**
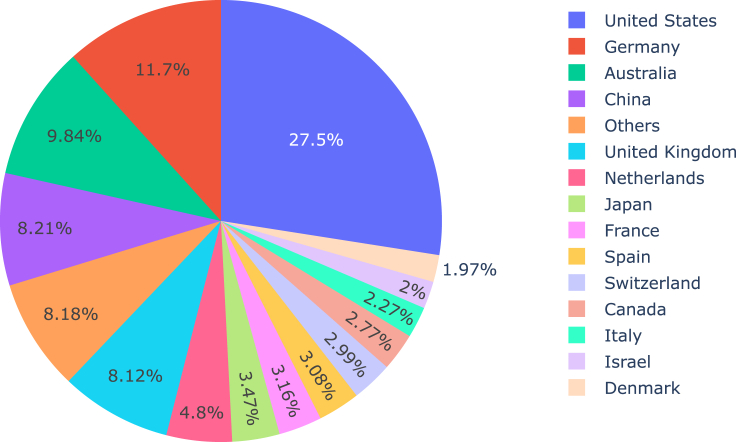
Country of origin of work curated by the IEDB.

At a second level, our investigation invites additional research not only on differences in performance across different economic levels but also on the relationship between existing economic differences and the social and historical circumstances that have helped make way for these differences and that appear inconspicuously in other ways around the data. Researchers have shown that biases in ML are not always grounded on arbitrary circumstances or statistical inaccuracies but are at times predicated on historical social practices and institutions.^31^ In the case of the pHLA datasets, associated metadata show that the samples were collected primarily from countries with higher levels of income. Figure 4 summarizes the country of origin for the institutions that conducted the experimental essays curated by the IEDB. More than a quarter of studies originate from institutions within the United States, followed by more than 11% from Germany, 9% from Australia, and around 8% from China. This information gives reason to speculate about human bias driving data bias: as institutions in higher income countries are able to collect more data, differences between allele representations become present in the database, ultimately leading to algorithmic bias as seen in the difference in performance between populations with lower and higher incomes. More information on the practices on how data are collected worldwide and how more opportunities for populations in lower income countries can be granted could in turn help shape strategies for including further data from these populations.

The ultimate aim of this study is to highlight the issues of bias in the pHLA binding prediction workflow and to highlight that this bias relates to the inherent systemic and historical patterns against geographic populations of a certain economic status. From dataset collection to algorithm development, the identified bias is perpetuated by pan-allele models. We hope that our work leads the community in continuing to recognize sources of bias such as those we identify in this study. Once the sources of bias are acknowledged, they can be mitigated. For instance, Norori et al. propose addressing existing bias in healthcare applications through open science^36^; this can be achieved through data sharing, setting proper data standards, defining proper evaluation metrics that are common among studies, and promoting AI explainability. Many of these propositions have already been established in the immunoinformatics community. Databases like IEDB^40^ and AFND,^40^ among others, share pHLA data effectively. New pHLA binding prediction approaches are adopting explainability modules moving away from the black-box ML paradigm.^22^ Another interesting avenue for allele bias mitigation is to train personalized, per-patient models.^42^ However, more work can be done regarding data collection, where very few studies sample binding affinities for alleles from different geographic populations.^37^^,^^41^

### Limitations of the study

Note that we focus our analysis on the two highly regarded state-of-the-art prediction tools (NetMHCpan4.1 and MHCFlurry2.0). We chose these two tools because they are the most widely cited among a range of other pan-allele predictors. It has been reported that most of the pan-allele tools rely on the same source of IEDB curated experimental data for training and that their training datasets have a large overlap of content.^8^ We see this overlap in our analysis between the NetMHCpan4.1 and MHCFlurry2.0 datasets. In particular, BA datasets of the two tools almost entirely align (see red markers in Figures 1, S1, and S2) while the MS datasets show an overlap across most of the alleles (see blue markers in Figures 1 S1, and S2). The reported overlap enhances the representativeness of our analysis. Nevertheless, we acknowledge that many other pan-allele predictors lie beyond the scope of our current analysis, presenting an exciting avenue for future work. In addition, we make our evaluation pipeline open source. Authors of future tools can test the population coverage of their training datasets prior to training.

There is an opportunity for further research, not only of the differences between geographic populations in accordance with their income level but also of the differences within a single geographic population in accordance with different “ancestries” in that population. Based on the World Bank’s classification table, we have considered geographic populations in the USA as “high income.” However, the geographic population of the USA does not have a homogeneous “ancestry.” Instead, as pointed out previously, it is composed of different *ethnogeographic* populations, such as USA European, USA Hispanic, USA African American, and USA Asian. As Figure 4 shows, the datasets cover the USA European population more than they cover populations with other ancestries. These differences in turn correlate to differences in levels of income between different ethnic populations in the USA,^43^ as predicated on practices of colonialism and ethnic segregation.^44^

## STAR★Methods

### Key resources table

**Table:** 

REAGENT or RESOURCE	SOURCE	IDENTIFIER
**Deposited data**		

NetMHCpan-4.1 training dataset	Reynisson et al.^9^	https://services.healthtech.dtu.dk/suppl/immunology/NAR_NetMHCpan_NetMHCIIpan/
MHCFlurry2.0 BA training dataset	O’Donnell et al.^2^	https://data.mendeley.com/datasets/zx3kjzc3yx/3 (Data S3)
MHCFlurry2.0 AP training dataset	O’Donnell et al.^2^	https://data.mendeley.com/datasets/zx3kjzc3yx/3 (Data S5)
MHCFlurry2.0 PS training dataset	O’Donnell et al.^2^	https://data.mendeley.com/datasets/zx3kjzc3yx/3 (Data S6)
Pyke et al. evaluation dataset	Pyke et al.^41^	Table S1 and Table S5 (in original source)
AFND Database	Gonzalez-Galarza et al.^40^	http://www.allelefrequencies.net/

**Software and algorithms**		

R	R	https://www.r-project.org/
Python	Python Software Foundation	https://www.python.org
NetMHCpan-4.1	Reynisson et al.^9^	https://services.healthtech.dtu.dk/services/NetMHCpan-4.1/
MHCFlurry2.0	O’Donnell et al.^2^	https://github.com/openvax/mhcflurry
IEDB Population Coverage tool	Bui et al.^39^	http://tools.iedb.org/population/download/
Analysis scripts	Original code	https://github.com/KavrakiLab/HLAequity

**Other**		

World Bank’s 2022–2023 "Country and Lending Groups" income classification table	https://datacatalogfiles.worldbank.org/ddh-published/0037712/DR0090755/CLASS.xlsx	N/A

### Resource availability

#### Lead contact

Further information and requests for resources should be directed to the lead contact, Dr. Lydia E. Kavraki (kavraki@rice.edu).

#### Materials availability

This study did not generate new unique reagents.

#### Data and code availability

• This paper analyzes existing, publicly available data. All of these datasets are exhaustively referenced in the key resources table. In addition, the processed data is available at: https://github.com/KavrakiLab/HLAequity.
• All the related code that is needed to reproduce the results of the study is available at: https://github.com/KavrakiLab/HLAequity.
• Any additional information required to re-analyze the reported data can be provided by the lead contact upon request.

### Method details

#### Mapping HLA alleles to geographic populations

We collect the distributions of alleles in different geographic populations from the Allele Frequency Net Database (AFND)^40^ (accessed May 2023). AFND collects data on the genetic variation of highly variable immune-related genes, including HLA genes. This type of data comes from more widely conducted population studies that are not specific to the pHLA binding prediction tasks.^40^ AFND has collected and curated data from more than 10 million people and classified them into more than 1600 population groups. Note that the AFND label of “population” contains both a geographic designation (the current country in which that population is found) and an ethnic designation (the “ancestry” of that population). For example, population labels for the USA appear in the AFND as USA Hispanic, USA Caucasian, USA Asian, USA African American, etc. However, for some populations the ethnic designation is missing or vague and the AFND states that the ethnic group designations are under revision and will be improved in the near future. For that reason, we focus our analysis on the geographic designation label as opposed to ethnic or ancestry-based labels. We refer to the population labels as “geographic populations”. The detailed description of the steps we took to download and clean the AFND data are described in the following paragraphs.

We query the AFND database for allele frequencies using the API http://www.allelefrequencies.net/hla6006a.asp for different loci (i.e., A, B and C). We format the allele names in the standard nomenclature as described by the IPD-IMGT^45^ keeping the information about the gene, the allele and the specific protein (i.e., format of A∗01:01). We map the population labels given by AFND to specific countries. We remove the duplicate entries for “population”-“allele” pairs. We perform sanity checks on the data to make sure that the allele frequencies per population add up to 1. As a result, we gather the cleaned population frequencies of different alleles across in csv files (one file per loci). We unify the per-loci population frequencies into a single larger file. We clean this data keeping only the populations that have information for each of the selected loci (i.e., A, B and C). This preprocessed AFND data is used as the background population frequency for calculating the population coverage of different datasets.

#### Classifying geographic populations by income

To better convey our findings on the existence of data and algorithmic bias in pHLA binding predictors, we group the geographic populations according to the income levels of the countries, and we perform a nation-based economic analysis. We acknowledge the relationship between current international and international economic differences and historical forms of ethnic segregation and oppression. As we explain in detail in the discussion section, by shedding light on existing economic differences between relevant geographic populations, we can then think more critically about these economic differences in relation to their historical complexities, including specifically on the history of colonization. In discussing the limitations of our study, our effort is precisely to invite more research that can help make these historical relationships clearer.

To classify the geographic populations based on their income level, we first identified the “country” appearing in each group’s label and then used the World Bank’s 2022–2023 “Country and Lending Groups” classification table to determine the income level for that country (World Bank information accessed at: https://blogs.worldbank.org/opendata/new-world-bank-country-classifications-income-level-2022-2023). The World Bank’s 2022–2023 “Country and Lending Groups” table classifies 217 countries around the world along four income levels (as defined by gross national income per capita in 2021). The four levels of income are low-income (∖$1085 or less), lower-middle-income (∖$1,086 to ∖$4,255), upper-middle-income (∖$4,256 to ∖$13,205), and high-income (∖$13,205 or more).

#### Examining data bias

To examine data bias, we analyze training datasets from two predictors that are widely used in the literature: MHCFlurry2.0^5^ and NetMHCpan4.1^3^. We choose these two state-of-the-art tools as they are most widely used and cited. A recent comprehensive study^8^ curated a list of pan-allele pHLA binding affinity predictors. We extract the number of citations for each of the tools from the Pubmed library and outline the number of citations in the following table. MHCFlurry2.0 and NetMHCpan4.1 are most widely cited in addition to being recent (see Table 1).

Note that both MHCFlurry2.0 and NetMHCpan4.1 gather data for training by querying the Immune Epitope Database (IEDB), where they find curated experimental data. We examine both the binding affinity (BA) portions (MHCFlurry2.0_BA, NetMHCpan4.1_BA) and the mass spectrometry (MS) portions (MHCFlurry2.0_MS and NetMHCpan4.1_MS) of the training datasets. The MS data can be either mono-allelic or multi-allelic. We refer to mono-allelic data as a definite peptide-HLA pair, while, in multi-allelic data, each peptide can potentially bind to up to six alleles. Deconvolution of the multi-allelic data is necessary in order to define the allele to which each peptide binds. To deconvolute the multi-allelic data, we used a binding affinity predictor (NetMHCpan4.1 or MHCFlurry2.0), and, for each peptide, we choose the allele to which the peptide has the strongest predicted binding affinity (out of six potential ones), thus converting multi-allelic data to mono-allelic data. All peptide pairs with a predicted binding rank of ≥0.5 are excluded, to remove peptides that do not bind to any of the designated alleles.^41^

We calculate the population coverage using the method devised by Bui et al. and implemented in the Immune Epitope Database (IEDB) tools.^39^ We use the AFND frequencies (see Section “mapping HLA alleles to geographic populations”) as ground truth allele frequencies of geographic populations. Population coverage has been used to estimate a portion of a population protected (“covered”) by a proposed peptide vaccine. In our study, instead of evaluating a quality of a vaccine, we are estimating the quality of the dataset. The inputs to the population coverage tool are peptide-allele pairs present in a dataset. We extract the PC90 metric calculated by the tool. PC90 corresponds to the number of data points in the dataset that covers 90% of the geographic population. To adequately compare differently sized datasets, we divide PC90 by the dataset size to get the scaled PC90 (sPC90). A lower sPC90 indicates that 90% of individuals in this geographic population are represented by a small portion of the dataset. High values of sPC90 indicate that 90% of individuals in this geographic population are represented by a large portion of the dataset. Ideally, the sPC90 of a dataset should be high and equal across different geographic populations.

#### Examining algorithmic bias

To test whether state-of-the-art binding prediction tools perform equally well among different alleles, we collected an independent dataset, found in Pyke et al.,^41^ which is not used in the training of state-of-the-art pHLA binding affinity predictors. This dataset is particularly valuable because it contains data on the alleles previously unseen in publicly available datasets. For example, HLA-A∗02:52, unique to this dataset, exhibits high frequency, (around 7%) in the Iranian Kurdish geographic population. The fact that these alleles were completely missing in the training phase of state-of-the-art pHLA binding affinity predictors mimics the case of testing the performance of a patient with a rare or unseen allele. The dataset consists of both mono-allelic and multi-allelic data points. We deconvolute the multi-allelic data as we did with the training datasets. Finally, as mono-allelic and multi-allelic data only contain binders, many non-binder peptides (decoys) need to be generated to evaluate binding prediction tools. We generate 500,000 decoys that are randomly selected from the human proteome for each peptide length found in the dataset (8-mers to 11-mers).^2^^,^^41^

### Quantification and statistical analysis

#### Accuracy metrics

To evaluate per-allele performance for state-of-the-art pHLA binding prediction tools, we employ the commonly used metrics Positive Predicted Value (PPV) and the Fraction Of Observed Peptides (FOOP).^2^^,^^41^ PPV for each allele is calculated by predicting binding scores for all positive peptide binders and for all the decoys generated from the human proteome. These predictions are then concatenated and ranked by order of strong to weak binding. We calculate the number of positives for each allele, $n_{a}$, and we take the top $n_{a}$ peptides from the ordering. The PPV for each allele, $PPV_{a}$ is equal to $\frac{\#hits\;from\;top\;n_{a}\;peptides}{n_{a}}$, taking a value between 0 and 1. The maximum $PPV_{a}$ value is equal to 1 when all top $n_{a}$ peptides in the ranking are binders, while the minimum $PPV_{a}$ value is 0 when all top $n_{a}$ peptides in the ranking are decoys. In short, PPV shows the likelihood that a pHLA with a high predicted binding affinity is truly a strong binder. For FOOP, we calculate the predicted rank of binder peptides within the 500000 negative sampled decoys. The binding affinity is predicted for the whole dataset and the position is each binder is noted as its rank. As an example, a rank of ≤0.1% is given to a peptide that is ranked within the first 500 decoys (0.1% of decoys), meaning that the peptide is a positive binder, and it is observed. FOOP is defined as the fraction of the positive pHLA instances that are predicted to bind in the top ≤0.1% of all the 500000 decoys (percentile rank ≤0.1%). A higher number, closer to 1, means that the number of strong binding peptides that are observed is much higher, showing the robustness of the model in identifying those strong binding peptides and separating them from the rest of the decoys.

#### Statistical significance

To evaluate the statistical significance of the differences of sPC90 coverage across population groups of different income levels (Figure 2) we use one-way ANOVA followed by the one-sided Kolmogorov-Smirnov (KS) test. We evaluate the KS statistic and the associated p values for all pairs of income levels (high, upper-middle, lower-middle, low) across all evaluated datasets (NetMHCpan4.1-BS).
