# Supplementary material for: HLAEquity: Examining biases in pan-allele peptide-HLA binding predictors
Source: iScience. 2023 Dec 2;27(1):108613. doi: 10.1016/j.isci.2023.108613 (PMC10770483; doi:10.1016/j.isci.2023.108613)
Supplement: Document S1. Figures S1–S3 [file mmc1.pdf]

iScience, Volume *27*

Supplemental information

HLAEquity: Examining biases in pan-allele peptide-HLA binding predictors

Anja Conev, Romanos Fasoulis, Sarah Hall-Swan, Rodrigo Ferreira, and Lydia E. Kavraki

SUPPLEMENTARY MATERIAL

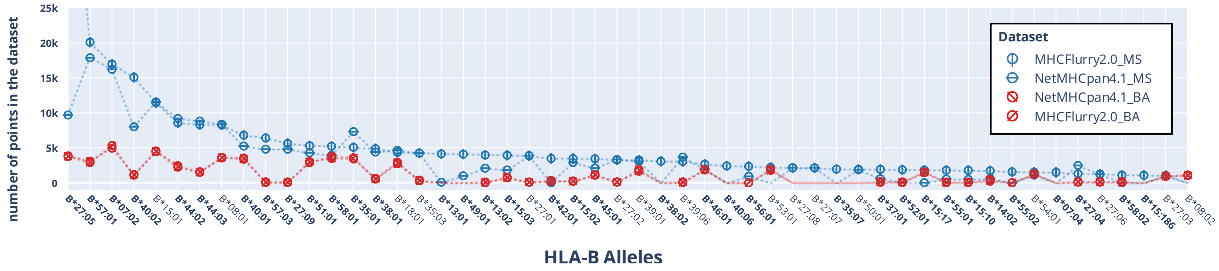

Figure S1 HLA-B allele frequencies in each of the training datasets. Related to *Figure 1*. Allele codes are indicated on the x-axis while the number of points in the dataset for each allele is indicated on the yaxis. Allele codes are bolded if the respective number of data points in MHCFlurry2.0_MS is higher than the number of data points in NetMHCpan4.1_MS.

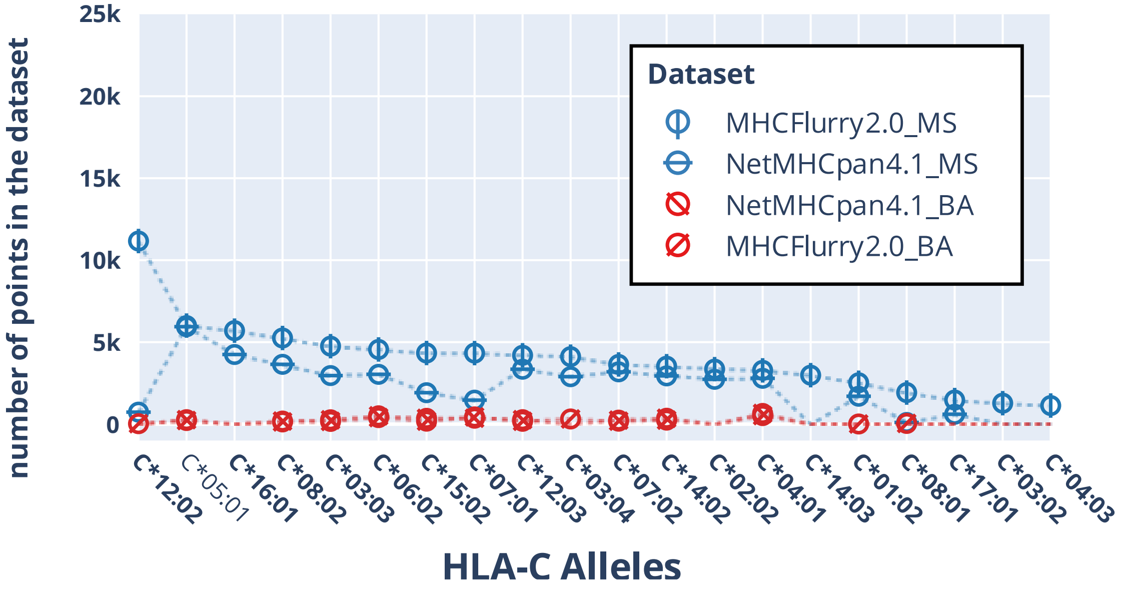

Figure S2 HLA-C allele frequencies in each of the training datasets. Related to *Figure 1*. Allele codes are indicated on the x-axis while the number of points in the dataset for each allele is indicated on the yaxis. Allele codes are bolded if the respective number of data points in MHCFlurry2.0_MS is higher than the number of data points in NetMHCpan4.1_MS.

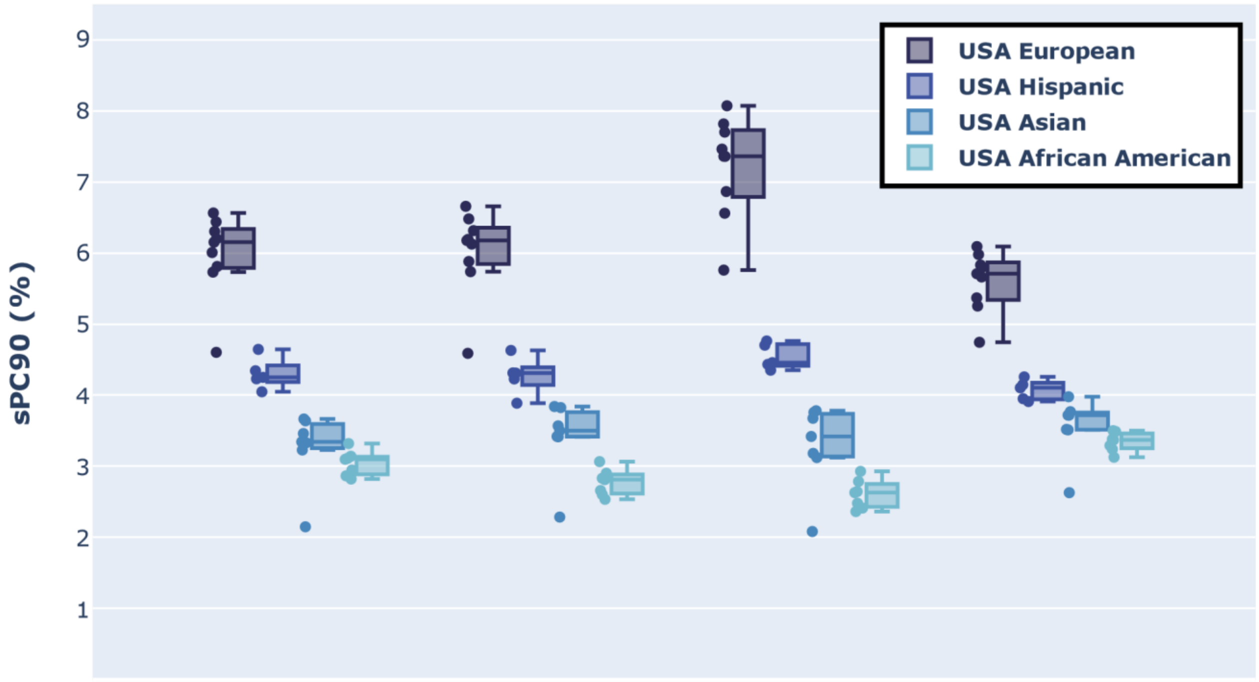

Figure S3. Scaled population coverage (sPC90) for the populations within the US. Related to *Figure 2*. Datasets are indicated on the x-axis. sPC90 values are indicated on the y-axis for each US population across the datasets. Each point corresponds to a particular population within the US and points are grouped based on the population ancestry.
